# How do alliances trade arms? Political alliance networks and global arms transfers

**DOI:** 10.1371/journal.pone.0282456

**Published:** 2023-03-01

**Authors:** Chelsea C. Chou, Chih-Sung Teng, Hans H. Tung

**Affiliations:** 1 Graduate Institute of National Development, National Taiwan University, Taipei, Taiwan; 2 Department of Political Science, National Taiwan University, Taipei, Taiwan; Pohang University of Science and Technology, REPUBLIC OF KOREA

## Abstract

The arms trade has come to the center stage of the international political economy. Yet only a few quantitative studies have been conducted on the political economy of the arms trade. This paper focuses on the security rents shared by trading partners in determining their arms trade relationship. We argue that the mechanism of reciprocity is better understood from the perspective of an international alliance network. Because the rents are likely to be transferred to other related members in a community, when a state is engaged in an arms deal with another state, it takes into account not only the direct dyadic relationship but also the alliance community to which the other state belongs. Based on this understanding, we employ social network analysis (SNA) to identify the effect of the international alliance community on the arms trade. Our findings suggest that if two states have a tie in a political alliance network, they are also likely to have a tie in the arms sale network. Moreover, we also find that the alliance network is a strong predictor of bilateral arms sales. Being in the same alliance community encourages two states to trade more arms with each other.

## Introduction

The arms trade has come to the center stage of the international political economy more than ever nowadays. As economic trade globalizes, so does the trade in weapons [1]. Yet, to our understanding, only a few quantitative studies have been devoted to the political economy of the arms trade, and most of them focus on the impact of the arms trade on economic growth [2]. This paper aims to explore the factors affecting states’ behavior of arms trade. Specifically, we focus on “the possibility of reciprocity” (the security rents shared by trading partners) in determining the arms trade relationship. We argue that, unlike Akerman and Seim where the difference in political regime type among trading partners is the key indicator [3], the mechanism of reciprocity is better understood when considering the nature of the international alliance network. When a state (A) mulls over potential security rents of arms deals with another state (B), it takes into account not only how different B is in its regime type, but also the alliance community to which B belongs. This is because the rents are likely to be transferred to other related states within B’s community. Based on this understanding, we employ social network analysis (SNA) in this article to identify the impact of the international alliance community on the arms trade.

The literature on international relations has long recognized the importance of networks in international politics. Networks are different from other modes of organization, such as states, political institutions, or markets. In the literature, networks are sometimes viewed as organizations that help solve collective action problems. Keck and Sikkink for example [4], focus on the role of transnational activist networks in promoting international agreements. Recently, scholars have emphasized that networks not only facilitate the connection between members but also shape the members’ incentives within the community [5, 6]. For instance, the impact of network ties has proven to be critical in explaining states’ unwillingness to engage in military conflicts with each other [7]. We adopt this viewpoint about networks and focus on how the behavior of members in a given network is influenced by the extra-dyadic relationship in the community.

The paper is structured as follows. The next Section discusses the theory and hypotheses of alliance networks and their effect on the arms trade. We then describe the data, and present the results of our network analyses and gravity models of arms sales and alliance network. The last Section concludes.

### Theory and hypotheses: Arms transfers and alliance network

Arms transfers are widely believed to be a strong instrument for states to achieve foreign policy goals. In their study of the relationship between political regimes and arms trade, Akerman and Seim argue that states tend to trade arms within their *political* vicinity [3]. That is, democracies favor democracies in arms transfers and at the same time, autocracies trade more arms with other autocracies as well. The issue of polity is related to arms trade through two major mechanisms. First, students of arms races have informed us of the possibility that security externalities discourage arms exports [8, 9]. In other words, arms exports are likely to create negative impacts on exporters’ national security, especially when the target state is a potential enemy. If we take into account what the democratic peace theory tells us [10, 11], we will reach a conclusion that trading weapons with states with a similar regime type is less likely to backfire, because democracies are less likely to engage in wars with each other.

Second, democratic governments tend to transfer arms with other democracies because the trade may generate political rents domestically and internationally. At home, the electorate is likely to reward political parties that support a trade policy containing human rights and democratic conditions. Governments thus have every intention of trading arms with democracies. At the international level, exporting arms to similar states is useful for making friends internationally and consolidating strong international alliances. Accordingly, Akerman and Seim state that “the global arms trade network should reflect constellations of political allies” [3].

We agree with Akerman and Seim that the concerns about arms sales’ negative security externalities as well as positive political rents are the main driving forces for states to trade weapons within their political vicinity [3]. However, we propose that these mechanisms are better conceptualized when taking into account the international political alliance relationship and structure than focusing on each state’s own regime type. The importance of alliance has been emphasized by the literature on the arms race. Scholars find that during the Cold War, the military preparation in the Western Alliance served as a public good for the members. This was due to the fact that a state’s military capacity had spillover benefits for its friends and hence could enhance the alliance’s capability [12]. In the post-Cold War era, while the arms trade network has been decentralized [13], the possibility of security benefits shared by allies still exists. This implies that alliance linkages are a more direct measurement of the relationship between two states than their differences in regime type. Accordingly, we argue that states’ decisions on arms transfer are highly influenced by their alliance relationships.

At the same time, trading arms with allies may generate domestic and international political rents. It is not hard to imagine that the electorate would be against exporting arms to their adversaries. At the international level, because arms sales consolidate states’ friendships, even Akerman and Seim themselves, while not exploring the relationship between the arms trade and political alliance, claim that the arms sale network may be seen as a proxy for alliance linkages [3]. In other words, having an alliance tie may induce states to establish an arms sale relationship. Based on this reasoning, we create our first hypothesis.

#### Hypothesis 1: Direct allies are likely to trade more arms than non-allies

In addition to a direct dyadic alliance relationship, we also examine the impact of the alliance network. Focusing on the latter has theoretical implications. In the existing studies, dyadic relationships are emphasized in determining states’ arms transfer decisions. The issue of regime type, for example, is a measurement of the direct difference between two states. In this paper, we argue that an *indirect* alliance relationship is highly associated with the flow of arms. Security and political rents generated by arms sales between two states are easy to be transferred to other members in both states’ alliance communities. States are thus likely to consider these indirect ties when making arms trade decisions. Focusing only on the direct relationship between two states overlooks the interdependence of international alliance networks. To address the problem, we use social network analysis to expose the impact of these indirect alliance linkages. To our knowledge, there has not been any study on the role of extra-dyadic alliance ties in determining states’ arms sale behavior.

In his research on economic trade, Haim provides an operationalized measurement to detect indirect alliance relationships [14]. He finds that higher levels of economic trade usually take place when they are in the same *alliance community*. When two states have a common ally, even if they have no direct dyadic alliance relationship, we may still say that these two states have an *indirect* alliance relationship. When allies become connected, we can identify integrated alliance communities. Haim created the alliance community variable to capture the direct dyadic and indirect alliance relationship between two states as well as the alliance community blocs [14]. It includes the intertwined alliance relationship among all connected states. Within an alliance community, the security and political rents generated by arms sales may multiply for its members. This critical role of communities in determining the dynamics of international relations has been recognized by many scholars in the field. In the study of the likelihood of war, for example, Lupu and Traag find that members of the same trade community are less likely to initiate military conflicts with each other and, more wars are found between members from different trade communities [7]. We employ the similar logic of shared community to examine states’ arms transfer behavior and formulate the hypothesis as follows.

#### Hypothesis 2: States in the same alliance community are likely to trade more arms than states from different alliance communities

In addition to the alliance community variable, which treats every state within a community as an equal partner, each state’s position in the network may affect the likelihood of economic trade [14]. Some states are in a core position in maintaining the security of the entire alliance community. In our arms sale case, trading arms with core states will also generate more security rents for all members within the same community. Accordingly, states will have more incentives to trade arms with the core states.

#### Hypothesis 3: A state is likely to trade more arms with core states than non-core states within its own community

In the next section, we present our data to test these hypotheses. We investigate the impact of the alliance network on arms trade through two steps. First, unlike much of the existing study which only examines a single network at a time, we compare the international political alliance network with the global arms trade network to see if the two networks have similar characteristics. Second, based on the gravity model illustrated by Anderson [15], we estimate the following augmented gravity model of the relationship between the alliance network and bilateral arms trade:

$$T_{ij}=\Omega^{\beta }\times \left(\frac{GDP_{i}^{\gamma_{1}}GDP_{j}^{\gamma_{2}}}{D_{ij}^{\gamma_{3}}}\right)$$

where *T_ij_* denotes the volume of arms trade between countries *i* and *j*, *GDP_i_* is the GDP of countries *i*, $D_{ij}^{\gamma_{3}}$ is the distance between countries *i* and *j*, and Ω is a matrix of our variable of interest, political alliance, as well as other related controls. The model is then linearized by taking a logarithmic transformation:

$$\ln\;T_{ij}=\ln\;\Omega \beta +\gamma_{1}\ln\;GDP_{i}+\gamma_{2}\ln\;GDP_{j}-\gamma_{3}{ln\;D}_{ij}$$

## Research design

### Data

We acquire data on arms trade volume from the Arms Transfers Database of the Stockholm International Peace Research Institute (SIPRI). The data time covers from 1951 to 2003. All the data are dyadic. The SIPRI data contain all international transfers of major conventional weapons and are widely used by scholars in the field. We collect all states’ available total bilateral arms exports for each year during the Cold War and the post-Cold War eras. To be consistent with previous literature, we exclude rebel groups from the sample.

On the other hand, alliance data are obtained from the Alliance Treaty Obligations and Provisions (ATOP) dataset [16]. The ATOP data consist of three kinds of alliance relationships: defense pacts, neutrality or non-aggression treaties, and ententes. All of them are regarded as political ties between states.

### Social network analysis

**Fig 1:**
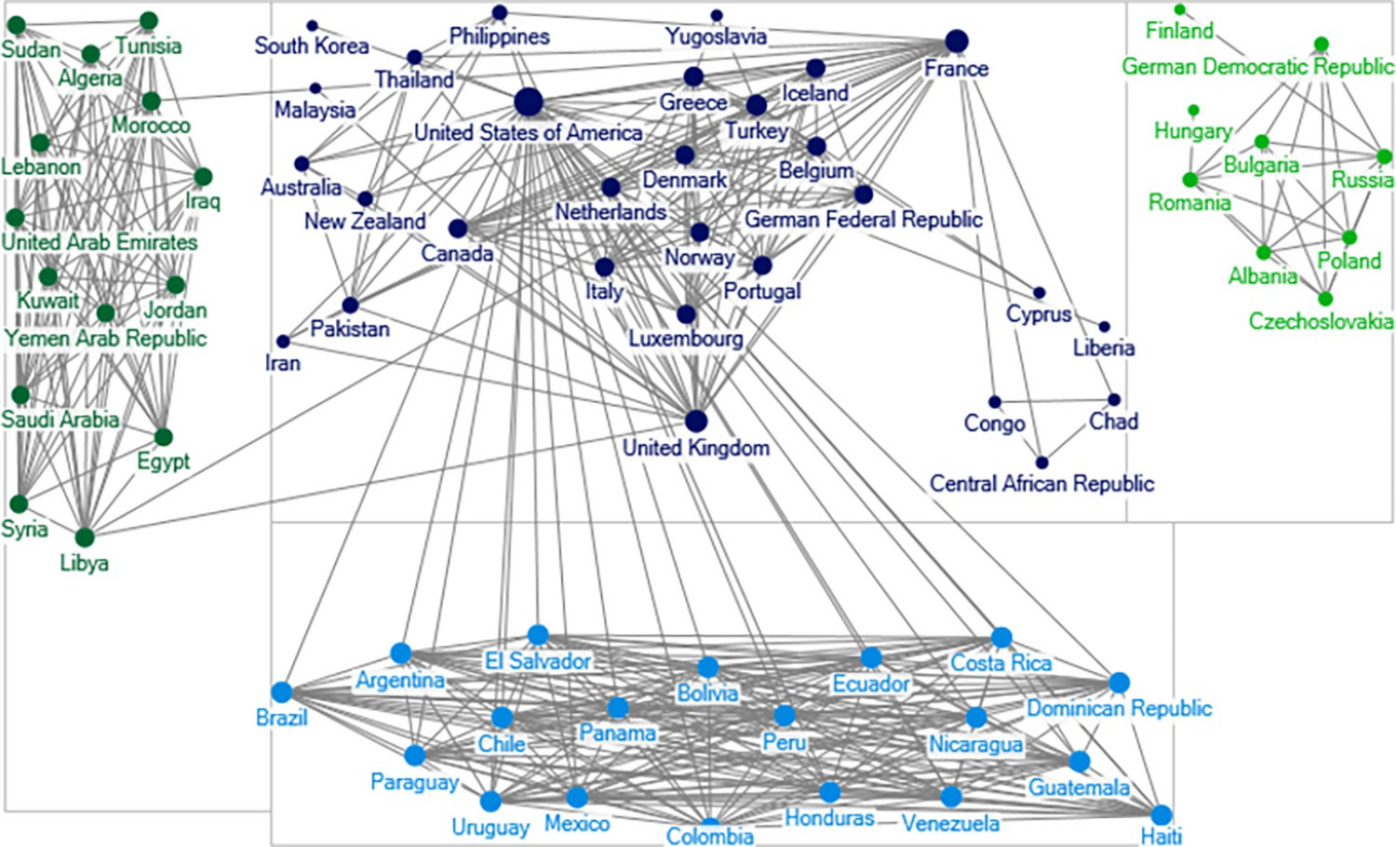
Political alliance network in 1965. A node is a state name. Ties connecting nodes represent political alliance relationship. The lines are undirected. Greater size of the notes indicates that the states have more political alliance ties.

**Fig 2:**
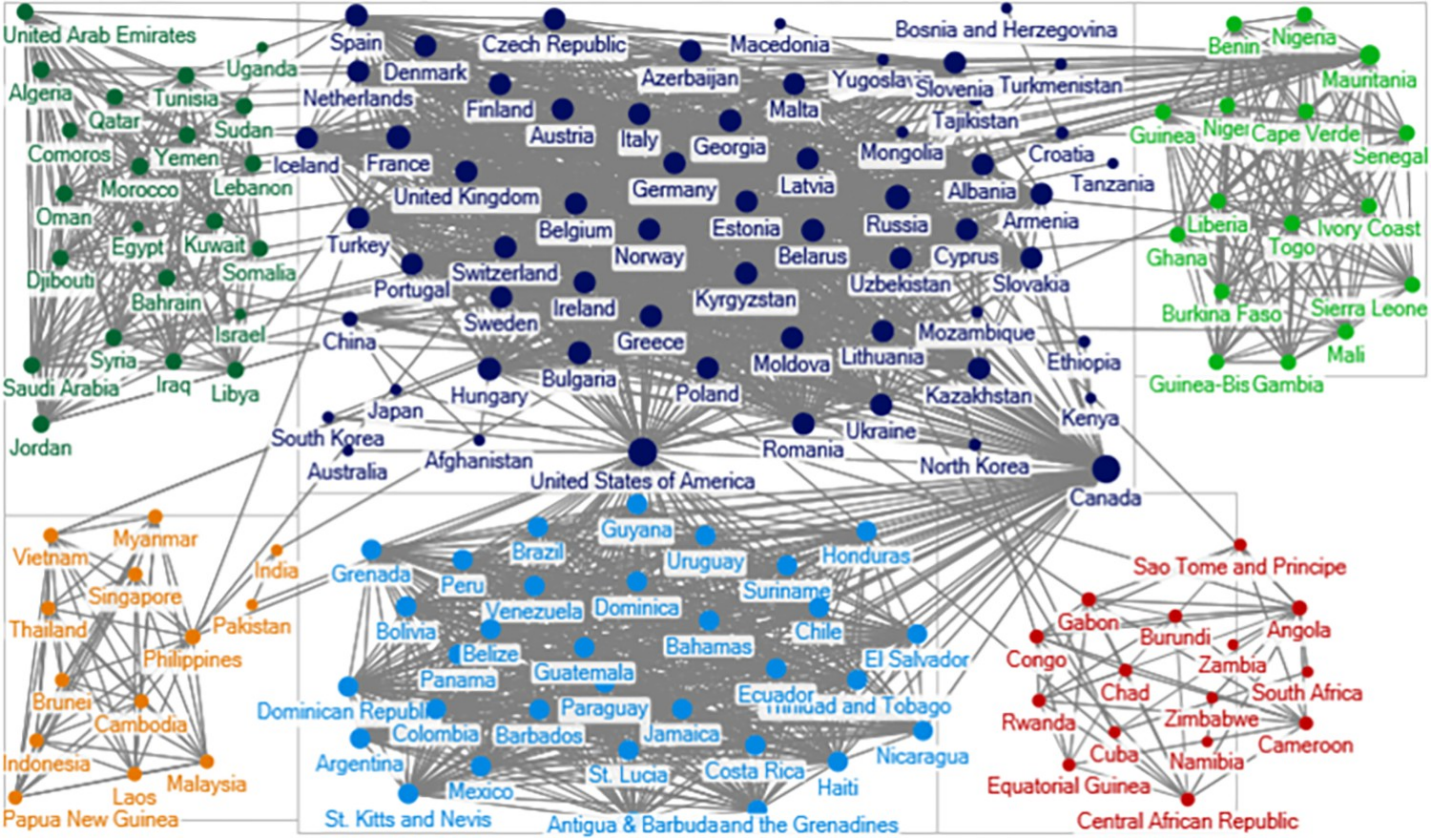
Political alliance network in 2000. A node is a state name. Ties connecting nodes represent political alliance relationship. The lines are undirected. Greater size of the notes indicates that the states have more political alliance ties.

The goal of social network analysis (SNA) is to explore the structural features of a set of interrelated actors [17]. Networks are often graphs consisting of nodes and lines. In our analysis, the unit of analysis is the alliance line connecting two states. Based on the ATOP alliance data, we draw the alliance network. In our graph, each state is a node, and each political alliance is a line connecting the two nodes. To save space, we show the global political alliance networks during the Cold War in 1965, and the post-Cold War in 2000 in Figs 1 and 2.

Comparing Figs 1 and 2, we see that the political alliance network in 2000 is much denser than that in 1965. In 1965, the Western and Soviet communities are clearly visible, and there isn’t a single tie that connects these two blocs. In 2000, all the communities have some ties with each other and the networks are much more intertwined.

**Fig 3:**
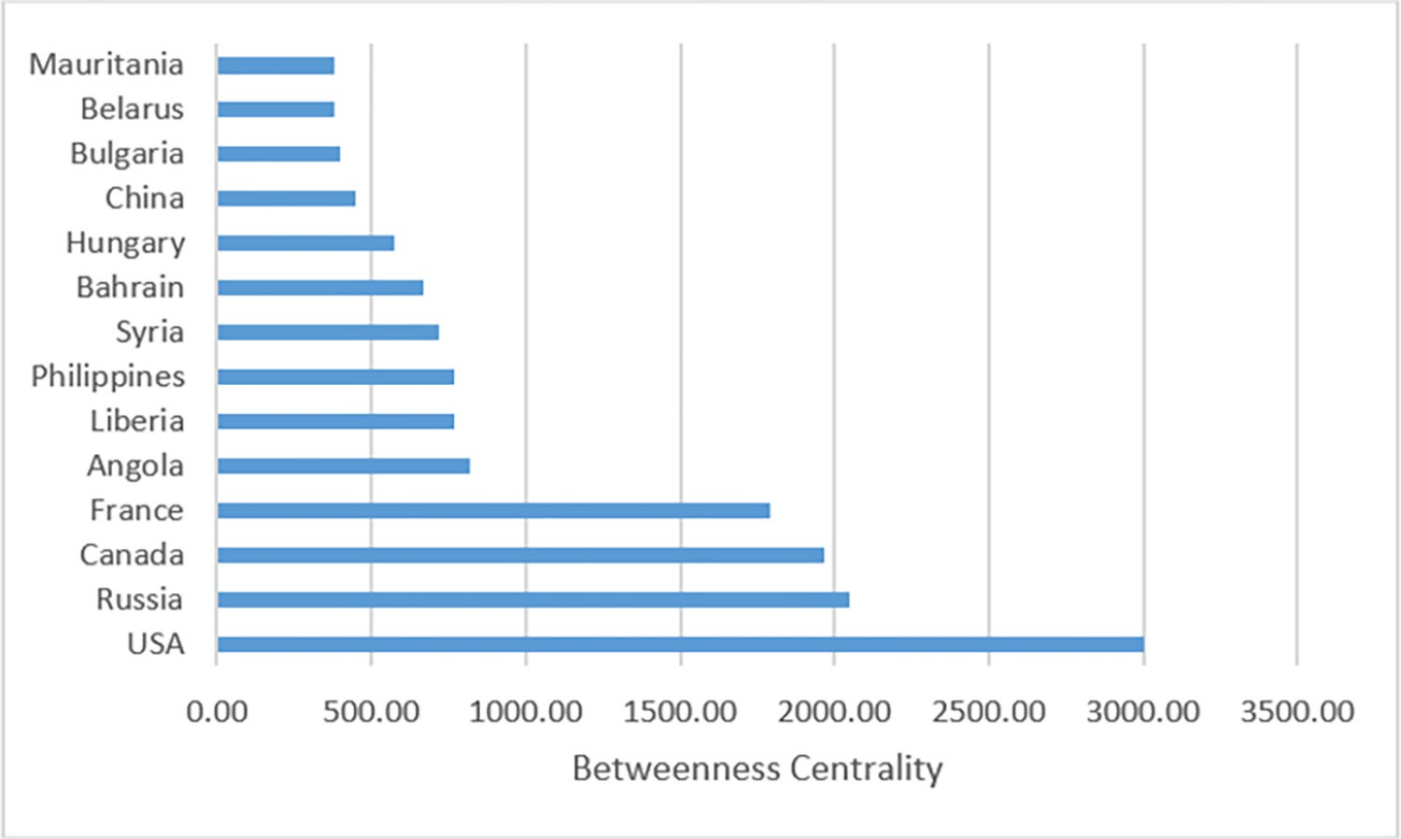
Betweenness centrality of selected states in the 2000 political alliance network. The first 14 states with a high degree of betweenness centrality are listed.

In addition, we calculate the betweenness centrality to find out the core states in a given political alliance community. Betweenness centrality measures the degree to which a node bridges the gaps between other nodes within a community. It is constructed by summing the number of shortest paths from all nodes to all others that pass through a certain node in a given year [18]. A higher number of betweenness indicates that the state has more control over resources and information in a given community. Fig 3 shows the betweenness centrality of states with the highest scores in the 2000 political alliance network.

**Fig 4:**
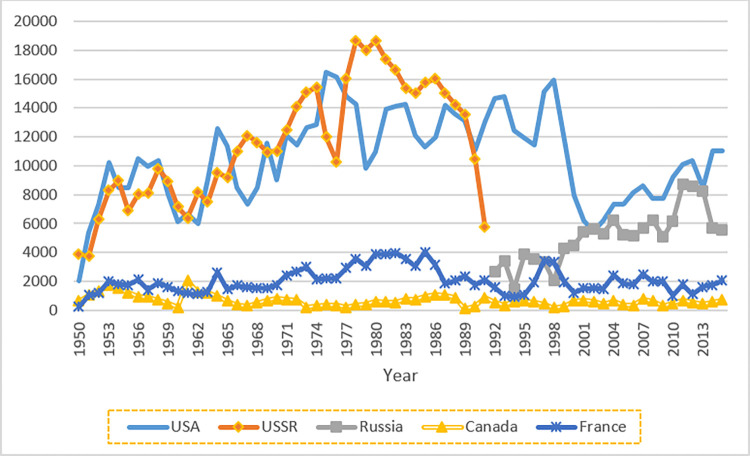
Total arms sale value by year of states with a high degree of betweenness centrality in the 2000 political alliance network, 1950–2015. The value includes exports from each state as well as imports to the state. Unit: US $m.

From Fig 3 we see that in 2000, the most critical states in forming political alliance communities are the USA, Russia, Canada and France. Below in Fig 4 we compare the total arms trade value (including imports and exports) of these states.

Fig 4 shows that the degree of betweenness centrality is somehow related to the arms sale value. The most critical state USA marks as the one with the highest arms sale value. Russia marks as the second one in both Figs 3 and 4, while the order between Canada and France in betweenness does not perfectly reflect the order in total arms sale value.

**Fig 5:**
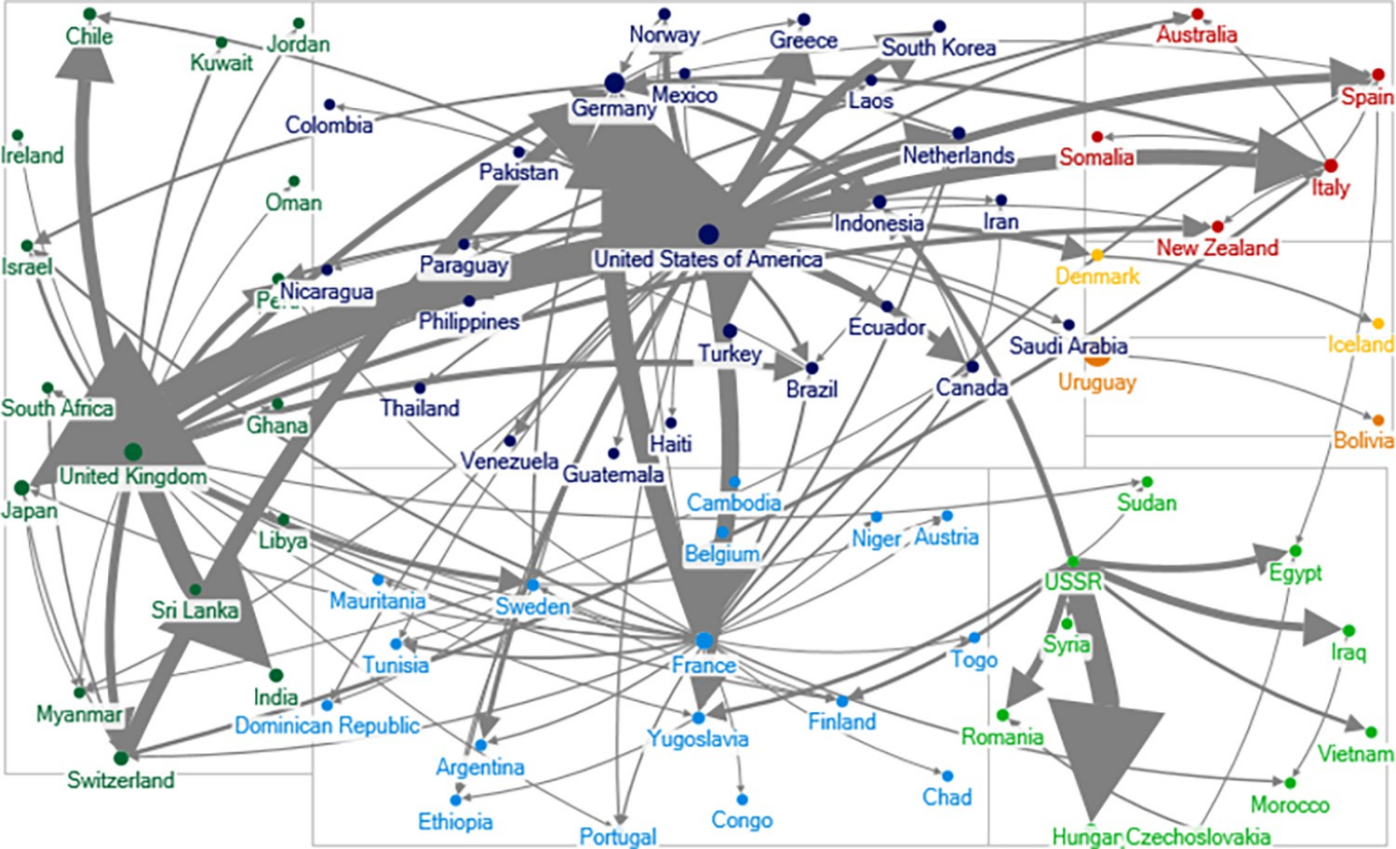
Arms export network in 1960. The lines are directed arrows and run from suppliers to recipients. The width of the line is related to the export volume.

**Fig 6:**
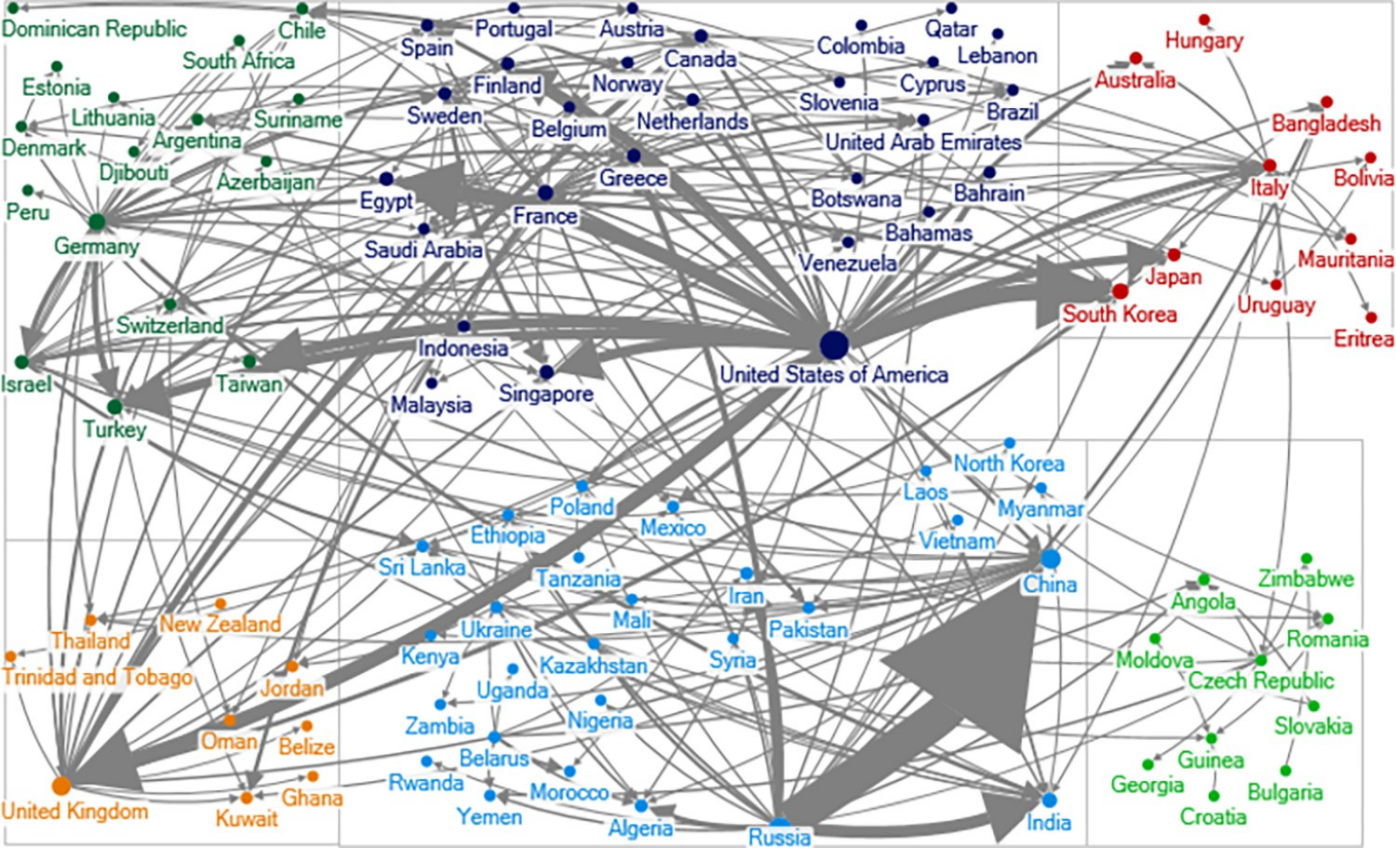
Arms export network in 2000. The lines are directed arrows and run from suppliers to recipients. The width of the line is related to the export volume.

After drawing the alliance network, we now turn our focus to the arms sale network. Figs 5 and 6 display the arms sale network during the Cold War and post-Cold War era.

From these two graphs, we see that the international arms trade network has become more decentralized from the Cold War to the post-Cold War era. This confirms the findings in Kinsella [13]. To have a better understanding of the basic characteristics of these networks, we first compare the density between the two arms trade networks in different years to trace the evolution from the Cold War to the post-Cold War era. In SNA, the density measures the ratio of all ties to the number of possible ties. Accordingly, in the arms sale network, the density measures the number of arms sales as a share of all possible arms sales. The Bootstrap method helps test which one has the higher density [19].

Table 1 shows that the density value of the 1965 arms trade network is 0.009, which indicates that 0.9 percent of all possible arms sales are conducted during the Cold War. On the other hand, the density value 0.0045 of the 2000 arms trade network indicates that 0.45 percent of all possible arms sales are carried out during the post-Cold War era. Thus, the arms trade network in 2000 appears to be less connected than in 1965.

**Table 1:** Density comparison of the political alliance network and the arms trade network (in 1965 and in 2000).

Bootstrap paired sample T-test	In 1965	In 2000
Density of Alliance	0.1096	0.1547
Density of Arms Trade	0.0090	0.0045
Difference in density	0.1005	0.1502
Number of bootstrap samples	10000	10000
Classical standard error of difference	0.0038	0.0023
Bootstrap standard error of the difference (indep samples)	0.0139	0.0110
Bootstrap SE for the difference (paired samples)	0.0123	0.0104
Proportion of absolute differences as large as observed	0.0001	0.0001

In addition, Table 1 reports that the difference between the density of the 1965 political alliance network and the 1965 arms trade networks is 10%. The density of alliance ties is greater than the density of arms sale ties. On the other hand, in 2000, the density of the alliance network is 15% higher than that of the arms sale network. This reveals that alliance networks are denser in general than arms trade networks. In addition, the difference in density between the alliance and arms trade networks in 2000 is greater than in 1965. We see from the comparison that while the global alliance network becomes denser in the post-Cold War era than in the Cold War era, the post-Cold War arms trade network does not reflect the same growth rate of density as the alliance network. In other words, during the Cold War, the overall difference in density between the political alliance network and the arms sales network was smaller, but in the post-Cold War period, the difference between them becomes larger.

On the other hand, if there is a tie between two states in political alliance relations, is there likely to be a tie between them in arms trade relations? To answer this question, we check the correlation of ties between the two networks. Here we are interested in the occurrence of arms trade relationships, rather than the trading volumes. Accordingly, we examine binary relations of the arms trade to compare the arms trade network with the alliance network. Simple matching and the Jaccard coefficient are commonly-used measures for binary relations [19]. Tables 2 and 3 present the univariate and bivariate statistics of the correlation of ties between the political alliance network and the arms trade network during the Cold War and post-Cold War era.

**Table 2:** Univariate statistics of networks.

Network	Mean	Std Dev	SSQ	MCSSQ	Euc Norm	N of Obs
Alliance in 1965	0.110	0.312	801	713.230	28.302	7310
Arms trade in 1965	0.009	0.095	66	65.404	8.124	7310
Alliance in 2000	0.155	0.362	3838	3244.182	61.952	24806
Arms trade in 2000	0.004	0.067	111	110.503	10.536	24806

SSQ (Sum of Squares): Total amount of squares of the differences from the mean.

MCSSQ (Mean Centered Sum of Squares): SSQ normalized.

Euc Norm (Euclidean Norm): Length of the straight line between a pair of nodes.

N of Obs: Number of observations (dyads).

**Table 3:** Bivariate statistics of the political alliance network and the arms trade network (in 1965 and in 2000).

				In	1965						In	2000		
	Value	Signif	Avg	SD	P(Large)	P(Small)	Number of Permutations	Value	Signif	Avg	SD	P(Large)	P(Small)	Number of Permutations
Pearson Correlation	0.272	0.000	0.000	0.021	0.000	1.000	10000	0.157	0.000	0.000	0.010	0.000	1.000	10000
Simple Matching	0.899	0.000	0.883	0.009	0.000	1.000	10000	0.850	0.000	0.842	0.008	0.000	1.000	10000
Jaccard Coefficient	0.082	0.000	0.008	0.005	0.000	1.000	10000	0.029	0.000	0.004	0.002	0.000	1.000	10000

The results in Table 3 indicate support for Hypothesis 1. In 1965, the correlation is 27.2%, and the Jaccard is 8.2%, while the correlation in 2000 is reduced to 15.7% and the Jaccard is 2.9%. Moreover, there is an observed simple matching of 89.9% in the 1965 result and 85% in the 2000 result. This means that in 1965 during the Cold War if there is a tie between two states in one network, there is an 89.9% chance that there will be a tie between these two states in the other network. In 2000, the chance is 85%. These values are all significant at the 0.1% level. We thereby conclude that the dyadic political alliance relationship is strongly associated with the bilateral arms sale relationship.

## Estimation

After establishing the bivariate associations, we estimate gravity models of arms trade value in dyads to test the hypotheses. Our dependent variable is the natural log of total bilateral arms export value from state *Ex* to state *Im*. (In our later network analysis, state *Ex* is an ego and state *Im* is an alter.) Our first baseline model includes all the essential predictors of a gravity model, namely logged country-level GDP of both exporting and importing states, logged country-level population of exporting and importing states, and logged geographic distance between the two states. To control the autocorrelation in arms trade flow, we incorporate a lagged measure of the dependent variable.

In accordance with Akerman and Seim [3], our second model tests the impact of differences in regime types on the likelihood of arms trade. The regime type variable is measured by the joint democratic level of both countries in a trading dyad. The data is obtained from the Polity IV dataset. The “Polity-score” is an indicator designed to capture the regime authority on a 21-pont scale ranging from a hereditary monarchy (-10) to a consolidated democracy (+10). While Akerman and Seim regard any state with a positive Polity-score as a democratic regime [3], this is not the common practice among political scientists. In fact, while a Polity value ranging from −10 to 0 is an autocratic regime, one ranging from +1 to +7 is usually regarded as a partial democratic regime [20]. For most political scientists, a full democracy should at least have a Polity-score greater than 5. Accordingly, in our analysis, if both states in a dyad have a Polity-score greater than 5, it is coded 1, and otherwise it is 0. In addition, it is highly likely that if two states are in militarized interstate disputes (MIDs) with each other, they are unlikely to trade arms. Accordingly, we include the MID data from the Militarized Interstate Disputes dataset version 3.1 of the Correlates of War project. If both states in a dyad are engaging in military conflicts in a given year, it is coded 1, and otherwise it is 0.

We also add several additional variables that are thought to be correlated with arms sales. First, students of international trade find that foreign policy preferences between states may influence states’ willingness of trade [21]. This may also affect states’ decisions about arms sales. We use the similarity of states’ votes in the UN general assembly, namely the S-score compiled by Gartzke [22], to measure the distance of states’ preferences. Other controls include geographic contiguity, colonial history, common language, and common currency. One thing worth noting is that since arms trade is exempt from WTO rules, we do not include trade agreement related variables.

To test the impact of the political alliance relationship, the variable *dyadic alliance* is included to measure the existence of direct dyadic political tie between two states. This is a dummy variable. On the other hand, the variable *shared community* is adopted from Haim [14]. The variable is operationalized as a dummy variable measuring whether the arms exporting state and importing state in a dyad are both members of the same alliance community in a given year. More specifically, the Fast Greedy community detection algorithms are used to identify highly connected clusters of nodes that form alliance communities [23].

## Regression results

Consistent with Akerman and Seim [3], we test our hypotheses with OLS estimation throughout the analysis. The descriptive statistics, correlation matrix, and results of our models are presented in Tables 4–6 respectively. Model 1 is based on the gravity variables. Model 2 shows the consistent finding with Akerman and Seim that political regimes are significantly associated with bilateral arms trade value [3]. Models 3 through 5 include the alliance related variables.

**Table 4:** Descriptive statistics.

Variable	Obs	Mean	Std. Dev.	Min	Max
Lagged arms export (log)	7960	1.437	0.751	0	3.648
GDP.Ex (log)	7960	13.049	1.579	7.076	16.206
GDP.Im (log)	7960	10.728	1.989	3.790	16.206
Population.Ex (log)	7960	4.162	1.202	0.572	7.161
Population.Im (log)	7960	3.059	1.565	-2.360	7.161
Distance (log)	7960	8.444	0.933	5.081	9.881
Military conflicts	7592	0.009	0.093	0	1
Joint democracy	7952	0.451	0.498	0	1
Dyadic alliance	6733	0.503	0.500	0	1
Shared community	6733	0.475	0.499	0	1
Contiguity	7960	0.074	0.262	0	1
S-score	7097	0.375	0.380	-0.7	1
Common currency	7960	0.019	0.137	0	1
Common language	7960	0.169	0.375	0	1
Common history	7960	0.337	0.473	0	1
Betweenness.Ex (log)	7486	4.674	3.432	0	8.347
Betweenness.Im (log)	7139	1.738	2.591	0	8.347

**Table 5:** Correlation matrix.

	GDP.Ex (log)	GDP.Im (log)	Population.Ex (log)	Population.Im (log)	Distance (log)	Lagged arms export (log)	Shared community	Dyadic alliance	Military conflicts	Joint democracy	Contiguity	S-score	Common currency	Common language	Common history	Betweenness.Ex (log)	Betweenness.Im (log)
GDP.Ex (log)	1																
GDP.Im (log)	0.221	1															
Population.Ex (log)	0.674	-0.159	1														
Population.Im (log)	-0.041	0.606	-0.093	1													
Distance (log)	0.303	-0.015	0.269	0.163	1												
Lagged arms export (log)	0.243	0.159	0.317	0.116	0.118	1											
Shared community	-0.062	0.187	-0.014	0.052	-0.295	0.121	1										
Dyadic alliance	0.174	0.114	0.213	-0.052	-0.336	0.151	0.568	1									
Military conflicts	0.028	-0.027	0.037	-0.018	-0.033	0.001	-0.009	0.053	1								
Joint democracy	0.192	0.501	-0.053	0.212	-0.100	0.100	0.336	0.373	-0.032	1							
Contiguity	-0.068	0.113	0.022	0.051	-0.421	0.065	0.167	0.180	0.059	0.062	1						
S-score	-0.488	0.120	-0.300	0.015	-0.406	-0.082	0.348	0.204	-0.056	0.179	0.214	1					
Common currency	-0.031	-0.140	0.022	-0.117	-0.064	-0.068	-0.013	0.078	0.024	-0.037	0.014	0.018	1				
Common language	-0.013	0.014	0.005	0.049	0.080	0.022	0.125	0.081	0.010	0.124	0.174	0.008	0.023	1			
Common history	-0.087	-0.024	-0.074	-0.032	-0.087	0.002	0.044	-0.060	0.015	0.001	0.213	0.045	-0.005	0.460	1		
Betweenness.Ex (log)	0.455	-0.202	0.591	-0.126	0.200	0.258	0.006	0.232	0.046	-0.044	0.022	-0.340	0.065	0.067	-0.063	1	
Betweenness.Im (log)	-0.045	0.342	-0.077	0.385	-0.015	0.077	0.132	0.155	0.045	0.133	0.039	0.094	-0.029	0.132	0.009	-0.095	1

**Table 6:** Estimation results.

	Dependent variable: Arms transfer flows (export)					
	(1)	(2)	(3)	(4)	(5)	(6)
Lagged arms export (log)	0.727***(0.008)	0.729***(0.008)	0.713***(0.009)	0.712***(0.009)	0.709***(0.009)	0.706***(0.009)
GDP.Ex (log)	-0.023***(0.006)	-0.024***(0.006)	-0.024***(0.007)	-0.021**(0.007)	-0.023**(0.008)	-0.028***(0.008)
GDP.Im (log)	0.027***(0.004)	0.024***(0.005)	0.022***(0.005)	0.021***(0.005)	0.024***(0.006)	0.028***(0.006)
Population.Ex (log)	0.070***(0.007)	0.071***(0.007)	0.066***(0.008)	0.065***(0.008)	0.066***(0.009)	0.056***(0.009)
Population.Im (log)	-0.002(0.005)	-0.001(0.005)	0.000(0.005)	0.001(0.005)	-0.001(0.006)	-0.002(0.006)
Distance (log)	0.012(0.006)	0.013*(0.007)	0.030***(0.008)	0.032***(0.008)	0.033***(0.009)	0.029**(0.009)
Military conflicts		-0.091(0.059)	-0.114(0.062)	-0.108(0.062)	-0.122*(0.062)	-0.131*(0.062)
Joint democracy		0.026*(0.012)	0.010(0.015)	0.005(0.015)	0.006(0.016)	0.011(0.016)
Dyadic alliance			0.051***(0.015)	0.030(0.017)	0.026(0.017)	0.031(0.016)
Shared community				0.041**(0.016)	0.045**(0.016)	
Contiguity					0.054(0.029)	0.043(0.029)
S-score					-0.033(0.022)	-0.007(0.022)
Common currency					0.001(0.047)	-0.015(0.047)
Common language					0.006(0.019)	0.002(0.019)
Common history					0.003(0.015)	0.008(0.015)
Betweenness.Ex (log)						0.011***(0.002)
Betweenness.Im (log)						0.003(0.003)
Constant	0.023(0.065)	0.035(0.067)	-0.069(0.08)	-0.108(0.081)	-0.100(0.100)	-0.054(0.100)
Observations	7960	7584	6385	6385	6124	6124
R-squared	0.594	0.597	0.583	0.583	0.585	0.586

Standard errors within parenthesis.

* Significance at the 5% level.

** Significance at the 1% level.

*** Significance at the 0.1% level.

While joint democracy is significant in model 2, when model 3 includes the dyadic alliance variable, the coefficient on joint democracy is decreased and no longer statistically significant. The coefficient on dyadic alliance in model 3 supports Hypothesis 1 and confirms what we obtain from the simple matching and the Jaccard coefficient in 3. But when the alliance community variable is included in model 4, the coefficient for direct dyadic alliance tie is largely reduced in magnitude and no longer statistically significant. In Model 5, we included more control variables that are commonly thought to be correlated with arms sales. These include S-score (the distance of states’ preferences) and several common attributes, including whether the two countries share a border, a common currency, a common language, and a common colonial history. All the measurement of these common attributes is operationalized as dummy variables. After including these additional variables that may be associated with the arms trade, the two hypotheses remained supported. The coefficients on joint democracy and direct dyadic alliance ties also become smaller and are not statistically significant, but both the magnitude and the statistical significance of the coefficient on the shared community variable remain unchanged.

The results show strong support for Hypothesis 2. The association between alliance community and arms trade is positive and, shared alliance community is a strong predictor of the arms trade. Our study confirms the strategic nature of the political alliance and its impact on the arms trade. States are more realistic than we might have expected. Since all members of the alliance community are able to enjoy security rents, when a state trades its weapons, it will give more consideration to selling to members of its own alliance community. This is true even in a situation where the state has no direct dyadic alliance ties with the member of its community.

Model 6 tests Hypothesis 3. We run the model on the subsample where the suppliers and recipients are in the same alliance community. The result shows that the natural log of states’ betweenness centrality is significantly associated with arms sales for exporting states but not for importing states. This implies that arms trade ties are strengthened by the core exporting states. For those states who play the critical role in an alliance community, they are also the main actors building arms trade relationships by exporting higher volume of arms to members within their alliance community.

One may question our model for the possibility of endogeneity. Indeed, the arms trade relationship may increase the likelihood of alliance formation. To answer this, we run another model with the five-year lagged alliance relationship and the shared community variables. The new model produces similar results, and the independent variables of interest remain significant. Moreover, we estimate a logistic regression with the shared community as the dependent variable and lagged arms trade and all the controls on the right-hand side of the equation. Lagged arms trade here is statistically insignificant.

## Conclusion

Using the SIPRI data on the international arms trade, this paper illustrates how arms trade is influenced by states’ direct dyadic and extra dyadic relationships in the international society. We find that if two states have a tie in a political alliance network, they are also likely to have a tie in the arms trade network. This finding is consistent across the Cold War and post-Cold War eras. Direct allies are likely to trade more arms than non-allies.

In addition, based on Haim’s network analysis on economic trade [14], we employ the social network analysis and show that an alliance network is a strong predictor of a bilateral arms sale. Extra dyadic alliance ties affect states’ decision to export arms, even when the destination has no direct alliance ties with the arms supplier. Being in the same alliance community encourages two states to trade more arms with each other and, states from different alliance communities tend to trade less. Our social network analysis also tells us that the volume of the arms trade is higher when the arms supplier is a critical state in the recipient’s alliance community. This means that the critical states are also the major actors in building international arms trade relations.

These findings in our paper not only advance how we empirically understand the impact of alliances on the arms sale, but also contribute to the theoretical discussions about the role of indirect relationships in international politics. The bulk of the current literature focuses on direct alliance ties. Our study explores the indirect ties intertwined in an alliance community. In this sense, our research takes a step further than Akerman and Seim by addressing the role of indirect relations in the arms trade [3]. We argue that the security and political rents generated by arm sales are better conceptualized through these indirect ties because the benefits are reciprocal and likely to be shared by all the members. Moreover, it’s precisely owing to the fact that two states are both members of a political alliance network that positive security externalities are created and their dyadic tie is consolidated. Our method, the social network analysis, helps capture these complex relationships in a political alliance network. This paper demonstrates the importance of applying SNA in international relations.
